# New Appliances &c.

**Published:** 1911-03-25

**Authors:** 


### NEW APPLIANCES & THINGS MEDICAL

### THE CHELTENHAM NATURAL APERIENT WATER.

(The Cheltenham Corporation. London Agents, Messrs. Ingram and Royle).

This aperient water is prepared by the artificial concentration of the Chadnor Villa Well at Cheltenham, and is a rich sulphate water with a relative small percentage of chlorides and silicates. Waters containing such a large percentage of magnesium and sodium sulphate, together with a small amount of calcium sulphate, are very uncommon in England, although not rare on the Continent. Leamington and Melksham in Wiltshire are the only real rivals to Cheltenham, and their waters cannot easily be obtained. This concentrated water which come from Cheltenham may therefore be looked upon as one of the most important home productions of its kind, and it compares remarkably favourably with foreign and much more expensive waters. Cheltenham Natural is sold at the cheap price of one shilling per large bottle, and is a bitter water with no added unpleasant taste. When poured into a wine glass it is a bright and sparkling liquid, although it contains, of course, no gas. As an aperient water it is suitable in all cases in which a natural aperient is prescribed; it does not gripe, and it may be relied upon to give good results, as its composition is stable. Practitioners in this country cannot do better than give this water a trial. We feel sure that having tried it they will not easily forsake it to return to the much-advertised foreign aperient waters which have really very few advantages over this water and which are much more expensive. The dose is from two to five ounces.

### VITTEL WATER.

(E del Mar, 12 Mark Lane, London, E.C.)

Vittel is a pretty little watering place in the Vosges, and its waters somewhat resemble those of Contrexiville. The Source Salee water contains small percentages of calcium carbonate and of calcium and magnesium sulphate, and is a mild aperient. The Grande Source water contains smaller quantities of the same salts with the addition of a small percentage of magnesium sulphate, which give to the water a slightly bitter flavour. The Source Salee water, on the other hand, is entirely free from any unpleasant taste, and is a still table water of remarkably smooth flavour. Bottled in the form it is put on the market, with every precaution taken to prevent impurities from entering it, the water is one of the best examples of the weaker alkaline waters that resemble the Contrexiville water in quality—though, in our opinion, it surpasses the latter in flavour. It can be used daily without giving rise to any of the unpleasant effects which the constant use of other and more powerful saline water produce.

### "HERO LIQUEUR" WHISKY.

A. Dewar Rattray's Old Wine Stores, 188 Dumbarton Road, Partick, Glasgow.

The sample of whisky forwarded to us was found to be a finely-blended, fully-matured whisky, with a distinctive flavour that is in striking contrast to the thin type of liqueur whisky so often met with, without being in any way too pronounced. In our elaborate review of whiskies, their composition and dietetic uses, published in The Hospital for April 7, 1905, we specially emphasised the importance from a therapeutical standpoint of the taste of whisky. The only action upon digestion that whisky can have as compared with an equal quantity of equally dilute ethytic alcohol depends upon the reflex action that it exerts upon the digestive functions through the palate. It is easy to under-estimate the primary importance of that reflex action, and that is the reason, we do not doubt, why so often fat, flavoured, "gripping" whiskies are often chosen for invalids and convalescents. These latter require, much more than the healthy individual, a mild, well-matured whisky. Such a brand is the sample of Hero whisky forwarded to us, which may be regarded as a first-class whisky with pleasant flavour and fine aroma, specially suitable for invalids, and entirely free from harmful by-products. The price charged is very low considering the excellence of the brand. The whisky is sent by post (postage per bottle eightpence) from the above address, or may be ordered in case or quantity.

